# Inhibition of quorum sensing regulation and stress tolerance by *Rhodomyrtus tomentosa* extracts and rhodomyrtone as an alternative treatment for zoonotic pathogens

**DOI:** 10.14202/vetworld.2025.877-887

**Published:** 2025-04-19

**Authors:** Sukanlaya Leejae, Watcharapong Mitsuwan, Ozioma F. Nwabor, Supayang Piyawan Voravuthikunchai

**Affiliations:** 1 School of Languages and General Education, Drug and Cosmetics Excellence Center, Walailak University, Nakhon Si Thammarat, 80160, Thailand; 2 Akkhraratchakumari Veterinary College, Research Center of Excellence in Innovation of Essential Oil, and One Health Research Center, Walailak University, Nakhon Si Thammarat, 80160, Thailand; 3 Department of Public Health, Syracuse University, Syracuse, NY13244, USA; 4 Center of Antimicrobial Biomaterial Innovation-Southeast Asia, Faculty of Science, Prince of Songkla University, Songkhla, 90112, Thailand

**Keywords:** anti-quorum sensing, *Pseudomonas aeruginosa*, rhodomyrtone, *Rhodomyrtus tomentosa*, *Staphylococcus aureus*, stress tolerance

## Abstract

**Background and Aim:**

*Staphylococcus aureus* is a zoonotic pathogen with significant public health and economic implications. Its ability to tolerate environmental stress and regulate virulence through quorum sensing contributes to its persistence and pathogenicity. *Rhodomyrtus tomentosa* and its bioactive compound rhodomyrtone have demonstrated antimicrobial properties against Gram-positive, multidrug-resistant bacteria. This study aimed to evaluate the effects of these agents on quorum sensing inhibition and stress tolerance in *S. aureus* and *Pseudomonas aeruginosa*, providing insights into their potential as alternative antimicrobial strategies.

**Materials and Methods:**

The anti-quorum sensing activity of *R. tomentosa* extracts was assessed using *Chromobacterium violaceum* as a bioindicator. In addition, the effects on *P. aeruginosa* swarming motility were evaluated. Stress tolerance in *S. aureus* was examined by subjecting treated cells to acidic (pH = 5.0), alkaline (pH = 9.0), osmotic (7.5% NaCl), heat (43°C), and oxidative (1 mM H_2_O_2_) stress conditions. The survival rates were determined through colony-forming unit (CFU) counts following treatment with rhodomyrtone and ethanol leaf extracts.

**Results:**

The ethyl acetate fraction of *R. tomentosa* leaf extract exhibited the highest violacein inhibition, followed by the ethanol extract. At 256 μg/mL, these extracts permitted *P. aeruginosa* colony formation but inhibited its swarming motility. Regarding stress tolerance, no surviving *S. aureus* cells were detected under any stress condition after 3–6 h of treatment with 2 × minimum inhibitory concentration (MIC) (1 μg/mL) of rhodomyrtone. In addition, 4 × MIC (128 μg/mL) of the ethanol leaf extract inhibited pathogen survival under all tested stress conditions except for alkaline and oxidative stresses.

**Conclusion:**

The findings suggest that *R. tomentosa* extracts and rhodomyrtone effectively inhibit quorum sensing and stress tolerance, offering a promising alternative antimicrobial approach. These compounds could be utilized in veterinary medicine and food safety to mitigate zoonotic pathogen contamination and combat antibiotic-resistant infections.

## INTRODUCTION

Zoonotic diseases are a significant public health concern because they can be transmitted between animals and humans and often cause severe infections. *Staphylococcus aureus* is a major zoonotic pathogen that affects a wide range of species, including humans, livestock, and pets, leading to substantial morbidity and economic losses in animal production systems [1, 2]. This pathogen is notorious for its ability to develop resistance to antibiotics, which renders infections increasingly difficult to treat [3]. Furthermore, no effective vaccine is currently available, highlighting the urgent need for alternative treatment strategies [4].

One of the key factors contributing to *S. aureus* pathogenicity is its ability to regulate virulence through quorum sensing [5]. The accessory gene regulator system in *S. aureus* enables the pathogen to modulate toxin production, biofilm formation, and immune evasion, enhancing its adaptability and persistence in various hosts [6]. In addition, quorum sensing mechanisms in Gram-negative bacteria, such as *Pseudomonas aeruginosa*, regulate motility, antibiotic resistance, and biofilm formation, making these systems potential therapeutic targets [7, 8]. Disrupting quorum sensing pathways can attenuate bacterial virulence without exerting selective pressure for resistance, making it an attractive alternative to conventional antibiotics [9]. In addition to quorum sensing, *S. aureus* exhibits strong adaptability to environmental stressors, allowing it to survive extreme conditions such as pH, temperature, salinity, and oxidative stress [10]. This stress tolerance enhances survival in diverse environments, including food processing facilities, medical settings, and within the host immune system [11, 12]. The sigma B (*sigB*) regulatory network plays a crucial role in modulating stress responses in *S. aureus*, contributing to antibiotic tolerance and pathogenicity [13]. Targeting these stress response mechanisms may provide a novel approach to reduce bacterial survival under hostile conditions. Natural plant-derived compounds have gained attention as potential antimicrobial agents due to their broad-spectrum activity and low resistance development [14]. *Rhodomyrtus tomentosa*, a medicinal plant native to Southeast Asia, has been extensively studied for its antimicrobial properties. The active compound rhodomyrtone has demonstrated potent activity against methicillin-resistant *S. aureus*, vancomycin-intermediate *S. aureus*, and other Gram-positive pathogens [15, 16]. Furthermore, the mechanisms of action of rhodomyrtone, its safety profile, and its potential medical applications have been extensively investigated [17–21]. In addition, previous studies by Mitsuwan *et al*. [22], and Limsuwan and Voravuthikunchai [23] have reported that *R. tomentosa* extracts inhibit biofilm formation and quorum sensing-regulated virulence factors in various bacterial species.

Despite the well-documented antimicrobial properties of *R. tomentosa* and its bioactive compound rhodomyrtone against Gram-positive pathogens, their effects on bacterial stress tolerance remain largely unexplored. While previous studies have demonstrated their ability to inhibit biofilm formation and quorum sensing-regulated virulence factors, the extent to which these compounds impair bacterial adaptation to environmental stress conditions, such as acidic, alkaline, osmotic, thermal, and oxidative stress, has not been comprehensively investigated. Addressing this gap is crucial for understanding the full potential of *R. tomentosa*-derived compounds as alternative antimicrobial agents that do not promote resistance selection.

This study aims to investigate the effects of *R. tomentosa* extracts and rhodomyrtone on quorum sensing inhibition in *P. aeruginosa* and *Chromobacterium violaceum* while also evaluating their impact on stress tolerance mechanisms in *S. aureus*. By elucidating their potential to suppress bacterial virulence and stress adaptation, this research seeks to determine the feasibility of these compounds as alternative strategies for controlling zoonotic pathogens in veterinary and food safety applications.

## MATERIALS AND METHODS

### Ethical approval

All experiments were conducted in compliance with the biosafety regulations for scientific research at Walailak University, Thailand, and approved under Ref. No. WU-IBC-66-016.

### Study period and location

The study was conducted from October 2023 to August 2024. Samples of *R. tomentosa* were collected from Songkhla Province, Southern Thailand. The preparation of *R. tomentosa* extracts and the purification of rhodomyrtone were performed at the Department of Chemistry, Faculty of Science, Ramkhamhaeng University, Bangkok, Thailand. Antibacterial and quorum sensing inhibition assays were performed at the Bacteriology Laboratory of the Tropical Medicine Laboratory, the Research Institute for Health Sciences building, Walailak University, Nakhon Si Thammarat, Thailand.

### Preparation of *R. tomentosa* extracts and rhodomyrtone purification

The leaves, flowers, and fruits of *R. tomentosa* were collected from Singha Nakhon District, Songkhla Province, Thailand. The classified reference voucher specimen of *R. tomentosa* (NPRC0057) was obtained from the Faculty of Traditional Thai Medicine, Prince of Songkla University, Hat Yai, Songkhla, Thailand. The air-dried leaves, flowers, and fruits were ground in an electric blender and extracted with 95% ethanol for 72 h before being evaporated to dryness using a rotary evaporator under vacuum at 50°C. The leaf extract was further fractionated using chloroform, ethyl acetate, ethanol, and aqueous solutions, followed by solvent evaporation until complete drying. The resulting extracts were dissolved in dimethyl sulfoxide (DMSO) at a concentration of 200 mg/mL and stored at −20°C until use.

Rhodomyrtone, which is represented by pale yellow needles, was isolated from *R. tomentosa* leaves. The purity of the compound was confirmed through nuclear magnetic resonance, infrared, and mass spectrometry analyses, with spectral data published by Hiranrat and Mahabusarakam [24]. The compound was dissolved in 10% DMSO for antibacterial assays.

### Bacterial strains and growth conditions

The bacterial strains used in this study included *S. aureus* American Type Culture Collection (ATCC) 29213 for stress tolerance assays, *C. violaceum* Department of Medical Sciences Thailand (DMST) 21761 for quorum sensing bio-monitor, and *P. aeruginosa* Natural Product Research Center (NPRC) 08 for quorum sensing and swarming assays. All strains were cultured on tryptic soy agar (TSA; BD Difco, USA) at 37°C for 16–18 h and maintained in tryptic soy broth (TSB; BD Difco). Long-term storage was performed in TSB containing 20% glycerol at −80°C.

### Quorum sensing inhibition

#### Paper disk diffusion assay for violacein inhibition

The anti-quorum sensing activity of *R. tomentosa* extracts was assessed using *C. violaceum* DMST 21761. The bacteria were inoculated in TSB and incubated at 37°C for 16–18 h. The bacterial suspension was adjusted to 0.5 McFarland standards and spread onto TSA plates. Extracts were dissolved in DMSO at a concentration of 200 mg/mL, and 12.5 μL of each extract was loaded onto disks to achieve a final concentration of 2.5 mg/disk. The disks were placed on plates inoculated with *C. violaceum* and incubated at 37°C for 24 h. DMSO was used as the control. The inhibition zone was measured, with anti-quorum sensing activity determined by the inhibition of violacein production without bacterial growth inhibition.

#### Quantification of violacein production

Violacein production by *C. violaceum* was evaluated using a flask incubation assay [25]. *C. violaceum* DMST 21761 was cultured in TSB and incubated at 37°C for 16–18 h. The bacterial culture was adjusted to 1 × 10^8^ colony-forming units (CFU)/mL and added to Erlenmeyer flasks containing TSB supplemented with the extracts at final concentrations of 250, 500, and 1,000 μg/mL. The flasks were incubated at 30°C with shaking at 150 rpm. After 24 h, 1 mL of the culture was centrifuged at 2,739× g for 5 min to separate the violacein precipitate. The precipitate was dissolved in 1 mL of 100% DMSO, and 200 μL of the solution was transferred to a 96-well microplate. The absorbance was measured at 595 nm. We performed serial dilutions of the culture and plated it onto TSA to quantify viable bacterial colonies and compared it with the control (1% DMSO).

#### Swarming motility assay in P. aeruginosa

The effects of *R. tomentosa* ethanol extract and the ethyl acetate fraction on *P. aeruginosa* swarming motility were tested on TSA plates containing 0.5% agar. The ethanol leaf extract and ethyl acetate fraction were selected due to their high violacein inhibition activity. The medium was supplemented with extracts at final concentrations of 8, 16, 32, 64, 128, and 256 μg/mL. The control was 1% DMSO. An overnight bacterial culture was adjusted to 0.5 McFarland standard, and 10 μL was spotted onto swarm agar plates before incubation at 37°C for 24 h. Swarming inhibition was determined by measuring colony size relative to the control.

### Effects of *R. tomentosa* ethanol extract and rhodomyrtone on *S. aureus* stress tolerance

The stress tolerance of *S. aureus* ATCC 29213 was evaluated under various conditions, including acidic (pH = 5.0), alkaline (pH = 9.0), osmotic (7.5% NaCl), heat (43°C), and oxidative (1 mM hydrogen peroxide [H_2_O_2_]) stress. The bacteria were precultured in TSB at 37°C for 18 h and subsequently inoculated into Mueller-Hinton broth (MHB; BD Difco). The cultures were treated with rhodomyrtone at 1/2 × minimum inhibitory concentration (MIC) (0.25 μg/mL), MIC (0.5 μg/mL), and 2 × MIC (1 μg/mL), or with ethanol leaf extract at MIC (32 μg/mL) and 4 × MIC (128 μg/mL). Aliquots were collected at 0, 1, 2, 3, 4, 5, 6, 7, 8, 10, and 12 h after treatment and plated onto Mueller-Hinton agar (MHA; BD Difco) under the respective stress conditions. The control cultures were incubated under normal conditions at 37°C. After 16–18 h of incubation, CFUs were counted to assess bacterial survival.

### Statistical analysis

The statistical analysis for this study was conducted using the Statistical Package for the Social Sciences (IBM SPSS Statistics 26, IBM Corporation, Armonk, NY, USA). Descriptive statistics, including mean and standard deviation, were used to summarize the data for quorum sensing inhibition, violacein production, swarming inhibition, and bacterial survival under stress conditions. One-way analysis of variance was performed to compare the effects of different concentrations of *R. tomentosa* extracts and rhodomyrtone on quorum sensing inhibition, violacein production, and swarming motility. When significant differences were observed (p < 0.05), Tukey’s Honestly significant difference test was applied for *post hoc* multiple comparisons. Adjustments for multiple comparisons were applied using the Bonferroni correction where appropriate. Data are visualized using bar graphs for comparative analysis and survival curves for stress tolerance experiments.

## RESULTS

### Anti-quorum sensing activity of *R. tomentosa* extracts

**Figure 1:**
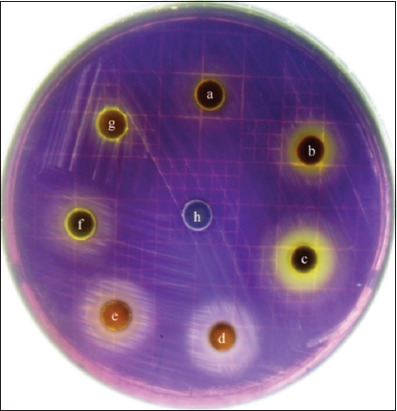
Inhibition of violacein production by extracts from different parts of *Rhodomyrtus tomentosa* (a) flower extract, (b) fruit extract, (c) leaf extract, (d) aqueous fraction of the leaf, (e) ethanol fraction of the leaf, (f) ethyl acetate fraction of the leaf, (g) chloroform fraction of the leaf, and (h) dimethyl sulfoxide (control) using *Chromobacterium violaceum* as a bio-monitor.

The bioactivity of *R. tomentosa* leaf extract and its pure compound rhodomyrtone was previously reported by our research group [15]. In this study, the anti-quorum sensing activity of the extracts was further evaluated against *C. violaceum*, a bio-monitor strain, using the paper disk diffusion assay as a preliminary test. The extracts from various plant parts, along with fractions of the leaf extract, were included in the analysis. The results demonstrated that all extracts exhibited anti-quorum sensing activity at a concentration of 2.5 mg/disk without inhibiting bacterial growth (Figure 1). The quorum sensing inhibition zones of the extracts ranged from 8.72 ± 0.04–11.57 ± 0.02 mm (Table 1). The largest ethanol fraction of the leaf extract was observed at 11.57 mm. In contrast, DMSO, which was used as the negative control, exhibited no quorum sensing.

**Table 1:** Quorum sensing inhibition zone of the extracts from different parts of *Rhodomyrtus tomentosa* using *Chromobacterium violaceum* DMST 21761 as a bio-monitor by disk diffusion assay (2.5 mg/disk).

Quorum sensing inhibition zone (mm)						

Ethanolic extract			Fractions of leaf extracts			
	
Flower	Fruit	Leaf	Chloroform	Ethyl acetate	Ethanol	Aqueous
8.72 ± 0.04	10.45 ± 0.15	10.82 ± 0.12	9.37 ± 0.13	9.67 ± 0.47	11.57 ± 0.12	10.15 ± 0.05

The control disk containing DMSO showed no anti-quorum sensing activity. DMSO=Dimethyl sulfoxide, DMST=Department of Medical Sciences Thailand

### Effects of *R. tomentosa* extracts on violacein production

**Figure 2:**
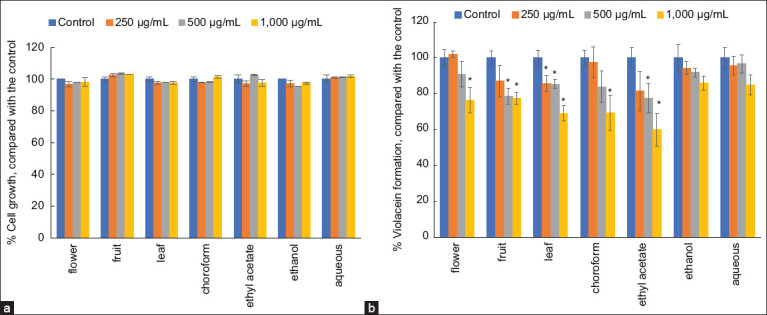
Effect of *Rhodomyrtus tomentosa* extracts at different concentrations on (a) the percentage of growth and (b) violacein produced by *Chromobacterium violaceum* Department of Medical Sciences Thailand 21761 compared with the control (1% dimethyl sulfoxide) (*significant difference; p < 0.05).

The results indicated that all extracts exhibited anti-quorum sensing activity, as demonstrated by the disk diffusion assay. The inhibitory effects of the extracts on violacein production by *C. violaceum* were quantified by measuring the optical density at 595 nm. The findings revealed that the reduction in violacein production was concentration-dependent (Figure 2b). The ethyl acetate fraction of the leaf extract exhibited the highest inhibition, with values ranging from 18.58% to 40.11% at 24 h (p < 0.05). At a concentration of 500 μg/mL, the ethanol fruit extract and ethyl acetate fraction significantly reduced violacein content compared with the negative control. However, none of the extracts at 250 μg/mL inhibited violacein production. It is noteworthy that the tested concentrations of the extracts did not inhibit bacterial growth compared with the control (Figure 2a).

### Effects of *R. tomentosa* extract on the swarming of *P. aeruginosa*

**Figure 3:**
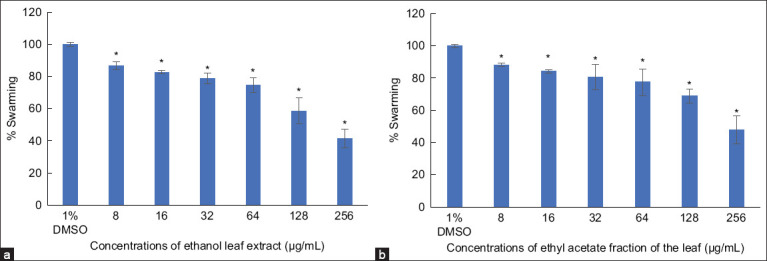
Effect of *Rhodomyrtus tomentosa* (a) ethanol leaf extract and (b) ethyl acetate fraction of the leaf extract at different concentrations on percentage of swarming produced by *Pseudomonas aeruginosa* Natural Product Research Center 08 compared with control (1% dimethyl sulfoxide) (*significant difference; p < 0.05).

Swarming is a significant virulence factor of *P. aeruginosa* and is regulated by the quorum sensing system. This study revealed that extracts from *R. tomentosa* reduced violacein production, which is regulated by quorum sensing. Therefore, we investigated the effects of leaf extract and the ethyl acetate fraction on *P. aeruginosa* swarming. The ethanol leaf extract and ethyl acetate fraction were selected for this study due to their high inhibitory effects on violacein production in *C. violaceum*. All concentrations tested (8–256 μg/mL) of the ethanol leaf extract (Figure 3a) and the ethyl acetate fraction (Figure 3b) significantly inhibited swarming in a concentration-dependent manner. At a concentration of 256 μg/mL, swarming inhibition was observed at 58.55% for the ethanol leaf extract and 52.11% for the ethyl acetate fraction.

### Stress tolerance of *S. aureus* treated with active agents

**Figure 4:**
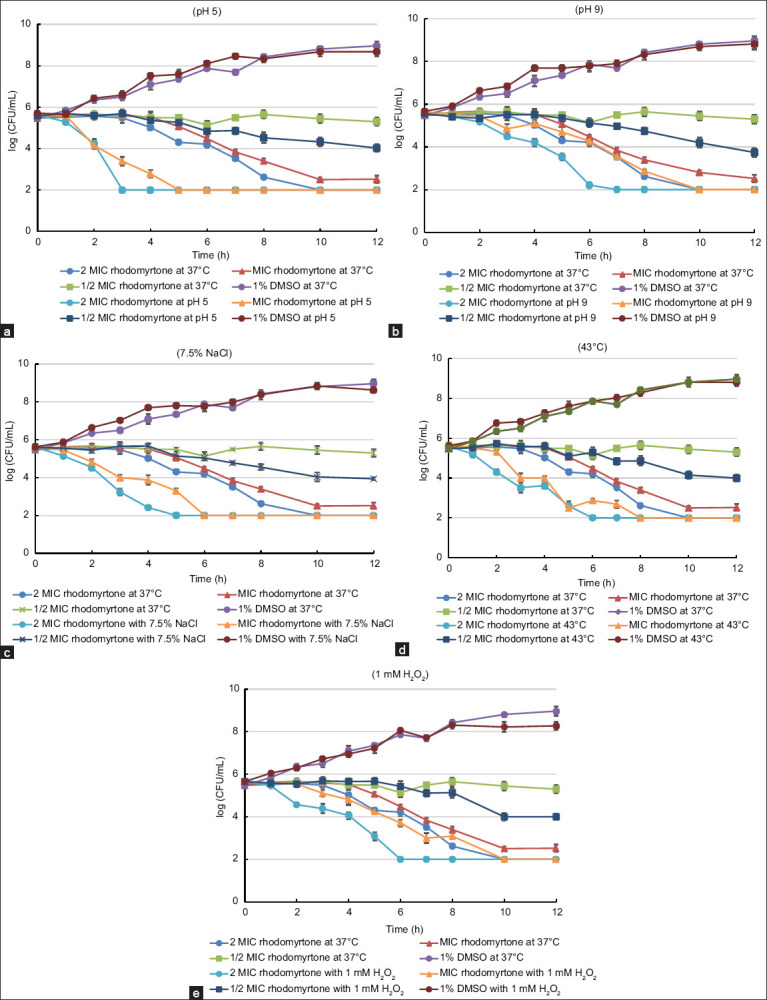
Growth of *Staphylococcus aureus* American Type Culture Collection 29213 on Mueller-Hinton agar adjusted to (a) pH 5.0, (b) pH 9.0, (c) 7.5% NaCl, (d) 43°C, and (e) 1 mM H_2_O_2_ after treatment with 2 × minimum inhibitory concentration (MIC), MIC, 1/2 × MIC of rhodomyrtone, along with 1% dimethyl sulfoxide. The treated pathogen was also cultured on normal Mueller-Hinton agar and incubated at 37°C as a control. Each symbol indicates the mean ± standard error for at least triplicate. The lowest detection threshold was 10^2^ colony-forming unit/mL.

To investigate the effect of stress conditions on *S. aureus* treated with rhodomyrtone and the ethanol leaf extract of *R. tomentosa*, the growth and survival of the treated organisms under various stress conditions, such as acidic and alkaline pH, high salt concentration, heat stress, and oxidative stress, were evaluated. The results indicated that the growth kinetics of the pathogens treated with 1% DMSO did not change under these stress conditions. In contrast, when *S. aureus* was tested for its survival under acidic conditions after exposure to MIC (0.5 μg/mL) and 2 × MIC (1 μg/mL) of rhodomyrtone for 3 h, the bacterial population significantly decreased by at least 2-3-log folds compared with the controls. Furthermore, no surviving cells were detected under stress after incubation with the active compound for 3–5 h (Figure 4a). Under alkaline challenge conditions, no surviving bacteria exposed to rhodomyrtone at 2 × MIC for 6 h were detected on MHA adjusted to pH 9.0 (Figure 4b). Interestingly, *S. aureus* treated with rhodomyrtone failed to grow under high salinity and heat stress conditions. Treatment with 2 × MIC of rhodomyrtone for 3 h resulted in an approximately 2-log reduction of viable cells on MHA supplemented with 7.5% NaCl and on MHA incubated at 43°C. Furthermore, no surviving cells were detected under both stress conditions after incubation with MIC and 2 × MIC of the active compound for 5–8 h (Figure 4c and d). For oxidative stress, a significant reduction in the viability of *S. aureus* treated with 2 × MIC of rhodomyrtone was observed, with at least a 2-log decrease within 3 h following treatment with 1 mM H_2_O_2_. In addition, no viable cells were detected under the stress condition after incubation with rhodomyrtone at MIC for 6 h (Figure 4e). This strong bactericidal effect is likely attributable to rhodomyrtone-induced membrane disruption, which enhances bacterial susceptibility to oxidative stress. Saising *et al*. [17] have shown that rhodomyrtone destabilizes the bacterial membrane, leading to increased permeability and leakage of cytoplasmic contents, including adenosine triphosphate and proteins. The loss of cellular integrity may explain the rapid cell death observed under oxidative stress conditions. Treatment with 1/2 × MIC (0.25 μg/mL) of rhodomyrtone inhibited bacterial growth after 12 h of exposure under all tested stress conditions (Figure 4a-e).

**Figure 5:**
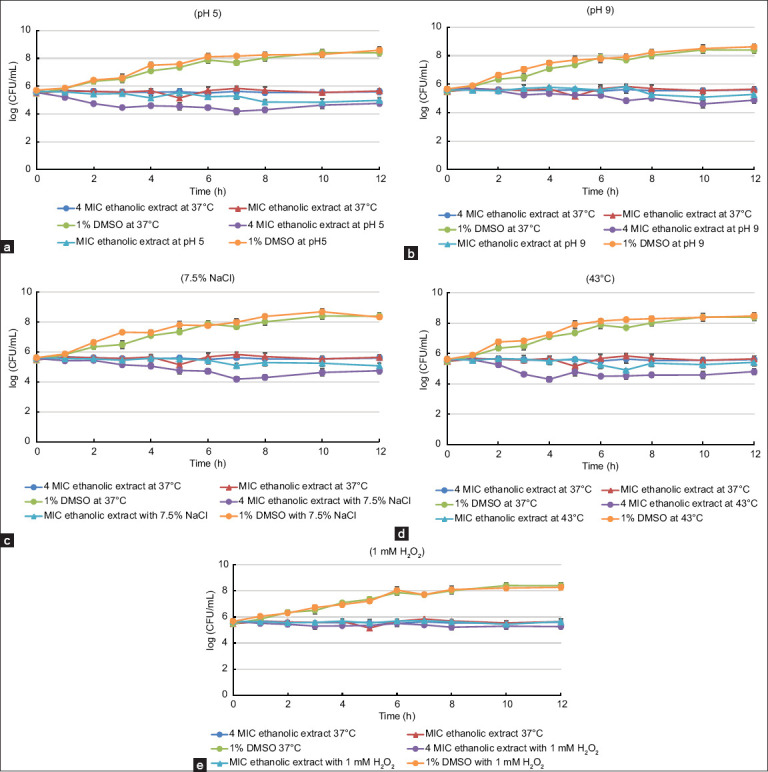
Growth of *Staphylococcus aureus* American Type Culture Collection 29213 on Mueller-Hinton agar adjusted to (a) pH 5.0, (b) pH 9.0, (c) 7.5% NaCl, (d) 43°C, and (e) 1 mM H_2_O_2_ after treatment with 4 × minimum inhibitory concentration (MIC), MIC of the ethanol leaf extract of *Rhodomyrtus tomentosa*, along with 1% dimethyl sulfoxide. The treated pathogen was also cultured on normal Mueller-Hinton agar and incubated at 37°C as a control. Each symbol indicates the mean ± standard error for at least triplicate. The lowest detection threshold was 10^2^ colony-forming unit/mL.

We also found that 4 × MIC (128 μg/mL) of the ethanol leaf extract slightly inhibited the survival of *S. aureus* under all tested stress conditions, except for alkaline and oxidative stresses. In contrast, the growth kinetics of the pathogen treated with the MIC of the extract remained unchanged under all tested stress conditions compared with the treated pathogen incubated under normal conditions (Figure 5a–e).

## DISCUSSION

Quorum sensing plays a crucial role in bacterial pathogenesis by regulating virulence factors, such as swarming motility, biofilm formation, and sporulation [26]. It has been implicated in chronic and recurrent infections, making it an attractive target for novel antimicrobial strategies. Natural products have been of particular interest due to their therapeutic effects in traditional medicine. Plant-derived quorum-sensing inhibitors have been reported to inhibit quorum sensing in bacterial pathogens [27, 28]. In addition, Limsuwan and Voravuthikunchai [23] have shown that *R. tomentosa* ethanol extract strongly inhibits quorum sensing, as all sub-inhibitory concentrations (0.24–7.81 μg/mL) significantly prevent biofilm formation in *Streptococcus pyogenes*. This study further explored the effects of different fractions of *R. tomentosa* leaf extract on quorum sensing in *C. violaceum*. The results indicated that the ethyl acetate fraction exhibited the highest violacein production inhibition, followed by the ethanol leaf extract. Based on these findings, we further assessed the effects of these extracts on the motility of *P. aeruginosa*. Swarming is a quorum sensing-regulated virulence factor in *P. aeruginosa*, a major zoonotic pathogen [29]. The inhibition of swarming through the inhibition of quorum sensing due to the inhibition of autoinducer-2 signaling, blocking N-acyl-homoserine lactone receptors, interspecies activity of alkyl quinolone signals, and enzymatic quenching of the signal [30]. This study demonstrated that both the ethyl acetate fraction and the ethanol leaf extract significantly inhibited swarming in a concentration-dependent manner. A previous study by Rajkumari *et al*. [31] has shown that triterpenoids such as betulin and betulinic acid, which are present in *R. tomentosa*, can inhibit the virulence of *P. aeruginosa* by targeting quorum sensing receptors. These findings suggest that *R. tomentosa* extracts interfere with quorum sensing mechanisms, making them a promising alternative to conventional antimicrobials. Furthermore, quorum sensing inhibition could also have practical applications in food safety because biofilm formation and toxin production in foodborne pathogens are often quorum sensing-regulated [32]. Disrupting this system, *R. tomentosa* extracts may help reduce bacterial contamination and prolong the shelf life of food products. Unfortunately, this study did not identify the pure compounds in the ethyl acetate fraction that exhibited anti-quorum activity.

*S. aureus* is widely distributed and exhibits remarkable adaptability to environmental stress, including exposure to acidic, alkaline, oxidative, heat, and osmotic conditions [10]. This stress tolerance significantly contributes to its pathogenicity by enabling survival under adverse conditions, such as in the host immune system or in food processing environments [11, 12]. *S. aureus* also possesses multiple defense mechanisms against oxidative stress, which are critical for its survival and ability to cause infections ranging from mild-to-severe. The findings of this study indicate that rhodomyrtone significantly impairs the ability of *S. aureus* to withstand oxidative, acidic, and alkaline stress compared with untreated controls. A previous study by Sianglum *et al*. [33] reported that rhodomyrtone downregulates *sigB*, a key stress response regulator in *S. aureus*. *SigB* plays a critical role in the adaptation of bacteria to stress by regulating the expression of various stress response genes [13]. The absence of *sigB* is associated with reduced bacterial resistance to heat, alkaline, and acidic stress conditions [34]. These results align with previous findings suggesting that rhodomyrtone disrupts stress adaptation pathways in *S. aureus*, increasing its susceptibility to environmental challenges.

*S. aureus* also tolerate high salt concentrations, which contributes to its ability to persist in marine environments and cause infection in fish. Therefore, the effects of rhodomyrtone on salt stress tolerance were evaluated. The results showed that rhodomyrtone-treated *S. aureus* exhibited significantly reduced survival in high-salinity conditions. This finding is consistent with previous study by Saising *et al*. [17], who demonstrated that rhodomyrtone rapidly reduces bacterial membrane potential, leading to ATP and cytoplasmic protein leakage. This membrane disruption may contribute to the reduced salt tolerance observed in this study. Notably, salt tolerance is a key survival factor for foodborne pathogens, allowing them to persist in processed and preserved foods, such as salted meat and seafood products. The reduction in salt stress tolerance by rhodomyrtone suggests its potential application in food preservation strategies to enhance microbial control and reduce contamination risks in high-salinity food products.

Infections caused by *S. aureus* occur in various animal species, including poultry [35]. The normal body temperature of chickens ranges from 40°C to 43°C, indicating that heat stress resistance is essential for the survival of *S. aureus* in poultry hosts. The present study examined the effects of heat stress on *S. aureus* following rhodomyrtone treatment. The results showed that rhodomyrtone-treated *S. aureus* failed to grow at 43°C. A previous study by Sianglum *et al*. [36] has reported that rhodomyrtone inhibits the synthesis of DnaK, a heat shock protein essential for bacterial survival under thermal stress. DnaK functions as a molecular chaperone that prevents protein mis-folding and aggregation under heat-stress conditions. Inactivation of *dnaK* has been shown to impair bacterial growth at elevated temperatures, as observed in other zoonotic pathogens such as *Brucella suis* and *Listeria monocytogenes* [37, 38]. These findings provide further evidence that rhodomyrtone interferes with bacterial stress response mechanisms, leading to increased susceptibility to heat stress.

## CONCLUSION

This study demonstrated that *R. tomentosa* extracts and rhodomyrtone effectively inhibit quorum sensing and impair stress tolerance in *S. aureus* and *P. aeruginosa*, highlighting their potential as alternative antimicrobial agents. The ethyl acetate fraction of *R. tomentosa* leaf extract exhibited the highest violacein inhibition, followed by the ethanol extract. At a concentration of 256 μg/mL, these extracts inhibited *P. aeruginosa* swarming motility while permitting colony formation. The evaluation of *S. aureus* stress tolerance revealed that no surviving cells were detected under acidic, osmotic, heat, and oxidative stress conditions after incubation with 2 × MIC (1 μg/mL) of rhodomyrtone for 3–6 h. In addition, 4 × MIC (128 μg/mL) of the ethanol leaf extract significantly inhibited bacterial survival under all tested stress conditions, except for alkaline and oxidative stresses. These findings suggest that targeting quorum sensing and stress tolerance mechanisms may offer a novel strategy for mitigating bacterial virulence and persistence, thereby reducing the risks associated with zoonotic pathogens in veterinary and food safety applications.

One of the strengths of this study is its comprehensive approach to assessing the dual antimicrobial mechanisms of *R. tomentosa* extracts, focusing on both quorum-sensing inhibition and bacterial stress tolerance. By utilizing well-established bioassays and stress adaptation models, the study provides robust evidence supporting the efficacy of plant-derived compounds in attenuating bacterial virulence without exerting direct selective pressure for antibiotic resistance. Furthermore, the study highlights the potential application of these natural compounds in controlling biofilm-associated infections and foodborne pathogens.

Despite these strengths, the study has some limitations. First, while the *in vitro* results demonstrate strong inhibitory effects on bacterial quorum sensing and stress tolerance, the lack of *in vivo* validation limits the direct translation of these findings to clinical or agricultural settings. In addition, the precise molecular mechanisms underlying the observed quorum-sensing inhibition and stress tolerance impairment remain unclear. Further studies should focus on identifying the active phytochemicals responsible for these effects and elucidating their mechanisms at the genetic and proteomic levels. Another limitation is the absence of toxicity assessments, which are crucial for determining the safety profile of these compounds before therapeutic application.

Future research should include *in vivo* studies in animal models to confirm the efficacy and safety of *R. tomentosa* extracts in real-world infection scenarios. Investigating their interactions with host immune responses and their potential synergistic effects with conventional antibiotics would provide valuable insights into their applicability as adjunct antimicrobial agents. In addition, efforts should be made to isolate and characterize the specific bioactive compounds responsible for quorum sensing inhibition and stress tolerance disruption. Expanding this research to other clinically relevant bacterial pathogens would further establish the broad-spectrum potential of these plant-derived antimicrobial agents.

Overall, this study provides compelling evidence that *R. tomentosa* extracts and rhodomyrtone offer a promising alternative antimicrobial strategy by targeting bacterial virulence and survival mechanisms. These findings contribute to the growing body of research on natural quorum-sensing inhibitors and stress response modulators, paving the way for the development of novel antimicrobial therapies that could help combat antibiotic-resistant zoonotic pathogens in both medical and agricultural settings.

## AUTHORS’ CONTRIBUTIONS

SL and WM: Conceptualization, experimental design, experimentation, data analysis, drafted, edited, and revised the manuscript. OFN: Experimental design, data analysis, and drafted, edited, and revised the manuscript. SPV: Conceptualization, experimental design, supervision, resources, and drafted the manuscript. All authors have read and approved the final manuscript.
